# Bacteremic sepsis leads to higher mortality when adjusting for confounders with propensity score matching

**DOI:** 10.1038/s41598-021-86346-4

**Published:** 2021-03-26

**Authors:** Lisa Mellhammar, Fredrik Kahn, Caroline Whitlow, Thomas Kander, Bertil Christensson, Adam Linder

**Affiliations:** 1 Division of Infection Medicine, Department of Clinical Sciences, Lund University, BMC B14, 221 84 Lund, Sweden; 2 Division of Anaesthesiology and Intensive Care, Department of Clinical Sciences, Lund University, Lund, Sweden

**Keywords:** Diseases, Medical research

## Abstract

One can falsely assume that it is well known that bacteremia is associated with higher mortality in sepsis. Only a handful of studies specifically focus on the comparison of culture-negative and culture-positive sepsis with different conclusions depending on study design. The aim of this study was to describe outcome for critically ill patients with either culture-positive or -negative sepsis in a clinical review. We also aimed to identify subphenotypes of sepsis with culture status included as candidate clinical variables. Out of 784 patients treated in intensive care with a sepsis diagnosis, blood cultures were missing in 140 excluded patients and 95 excluded patients did not fulfill a sepsis diagnosis. Of 549 included patients, 295 (54%) had bacteremia, 90 (16%) were non-bacteremic but with relevant pathogens detected and in 164 (30%) no relevant pathogen was detected. After adjusting for confounders, 90-day mortality was higher in bacteremic patients, 47%, than in non-bacteremic patients, 36%, *p* = 0.04. We identified 8 subphenotypes, with different mortality rates, where pathogen detection in microbial samples were important for subphenotype distinction and outcome. In conclusion, bacteremic patients had higher mortality than their non-bacteremic counter-parts and bacteremia is more common in sepsis when studied in a clinical review. For reducing population heterogeneity and improve the outcome of trials and treatment for sepsis, distinction of subphenotypes might be useful and pathogen detection an important factor.

## Background

Sepsis is considered a “life-threatening organ dysfunction caused by a dysregulated host response to infection” and detailed data on its cause is fundamental^1^.

Culture sampling is reported to only identify the causative organism in blood culture isolates (i.e. bacteremia) in 15–30% of the sepsis patients^2–7^. Additionally, 20–30% have a positive pathogen isolate from other locations. This means that at least 40% of sepsis patients are culture-negative or “sterile”^2,6,8,9^. The origin of this high rate of sterility is unknown. The low sensitivity of microbiological tests is believed to be one reason^10^. Another factor to be considered is if antibiotics have been administered prior to collection of microbiological cultures^11^. Other possible causes are viral infections, intermittent presence of bacteremia and non-infectious diagnoses misinterpreted as sepsis^12^. The only treatments in sepsis are either antimicrobial therapy or supportive care. In sterile sepsis, the treatment remains empiric with risk to be ineffective^13–16^. One can falsely assume that it is well known that bacteremia is associated with higher mortality. Only a handful of studies specifically focus on the comparison of culture-negative and culture-positive sepsis and have resulted in evidence both for and against bacteremia being associated with higher mortality^6,17–22^*.* In a large, prospective study by Phua et al. mortality was not higher among culture-positive patients in a multivariate analysis and Gupta et al. found higher mortality in culture-negatives, however antibiotic therapy preceding culture sampling was not included in the models^2,9^. Nannan Panday et al. took prior antibiotic therapy into account when retrospectively analyzing a prospectively gathered cohort, and found higher mortality among bacteremic patients^6^. In a large study based on data on septic shock patients from several prospective studies Kethireddy et al. found similar mortalities in culture-positive as in sterile sepsis patients.

There is no effective treatment addressing the sepsis reaction, possibly due to the heterogeneity in sepsis^23^. An endotype or subphenotype is a subset of a patient population defined by observable characteristics, distinguished from the population as a whole by natural history, disease manifestation and/or response to treatment^24^.

The aim of this study was to describe characteristics and outcome for Intensive Care Unit (ICU) patients with sepsis with bacteremia, pathogen-detected but non-bacteremia, and for sterile sepsis, while taking treatment with antibiotics at the time of culture sampling into account as a confounder.

We also aimed to identify subphenotypes of sepsis with culture status included as candidate clinical variables.

## Methods

The primary outcome is whether there are differences in mortality between bacteremic sepsis and non-bacteremic sepsis.

Secondary outcomes are differences between the groups with regard to severity of disease, site of infection, comorbidities, preceding antibiotic therapy and other immunomodulatory medications and if there are subphenotypes with clinically distinction on prognosis.

### Patient selection and data collection

Patients > 18 years admitted to the general intensive care unit (ICU) at Skåne University Hospital in Lund Sweden, between 2007 to 2014 and classified with the diagnosis sepsis or septic shock diagnosis according to treating ICU physician during the length of stay, were eligible for inclusion. Additionally, medical charts of all patients were reviewed by an infectious disease specialist (LM) for accuracy of diagnosis and all patients without infection diagnosis as a cause for organ dysfunction according to sepsis-3 (≥ Δ2 Sequential Organ Failure Assessment (SOFA) score) were excluded, see supplementary information for sepsis-3 definition^1,25^. The Definitions of Infection in the Intensive Care Unit of the International Sepsis Forum Consensus Conference, were applied with exception for demands on microbiological verification^25^. Further, at least one blood culture sampled between 48 h before and 48 h after ICU admission for each subject was required. Patients previously submitted to another ICU were excluded, as were re-admissions.

Patients were identified from the local quality registry (PASIVA Otimo Data AB, Kalmar, Sweden) and screened for inclusion. Date of admittance to and discharge from the ICU, mortality status at discharge from the ICU, survival days post discharge, SOFA scores, data on mechanical ventilation, renal replacement therapy and administration of vasopressors were also obtained from the local quality registry.

All cultures and other microbiological tests sampled within the 96-h time frame, further sampling before/after 96 h, antibiotic treatment within 48 h before first culture, source of infection, steroids, chemotherapy and immunomodulatory medications within 6 months before admission to ICU and comorbidities according to Charlson Comorbidity Index (CCI) were obtained through manual extraction from medical records.

All microorganisms in samples were assessed for sepsis-causing pathogen, colonization or contamination by an infectious disease specialist (LM). The microbial analyses were performed at the Clinical Microbiology Laboratory as part of clinical practice. For blood culture the BacT/Alert was used. A positive blood culture was defined as growth in blood culture of pathogens or growth in blood culture with the same possible skin contaminants in two or more cultures drawn from separate venous punctures.

Ethical approval was obtained from the the Lund University Ethical Review Board (decision number 2015/285). The Lund University Ethics Review Board waived the requirement for informed consent. All methods were performed in accordance with the relevant guidelines and regulations**.**

### Statistical analysis

Patients were categorized into bacteremic sepsis, pathogen-detected but non-bacteremic sepsis (i.e. had negative blood cultures but a positive isolate from another body location classified as the focus of infection), and sterile sepsis. Categorical variables are presented as frequencies and percentages and continuous variables as medians with 95% Confidence Interval (CI) and interquartile range (IQR). The groups were compared with Pearsons χ^2^ or Fisher’s exact test for proportions, as appropriate, while Mann–Whitney U test and Kruskal Wallis were used for comparison of medians.

We used logistic regression for propensity score matching to balance groups of bacteremic and non-bacteremic patients with potential confounders; age, Charlson comorbidity index, preceding antibiotic therapy and site of infection. We used “nearest-neighbour” matching without replacement at a ratio of 1:1 and accepted a propensity variable match tolerance of 0.01. χ^2^ or Fishers exact test was used to compare baseline categorical variables and Mann–Whitney U test for continuous variables.

In sepsis, where a formal definition is a construction, subphenotypes can be constructed out of observable characteristics. Latent Class Analysis (LCA), a machine-learning approach, is a statistical method to identify unmeasured class membership in order to derive subphenotypes in a non-biased way. In contrast to traditional regression analysis which finds association between variables and a known outcome, the LCA models create classes defined by variables without consideration of the outcome.

LCA was used to identify sepsis classes. The number of classes were tested for model fit, starting from one until additional classes did not improve the model fit. The model fit was estimated by Akaike Information Criterion (AIC), Bayesian Information Criterion (BIC), adjusted Bayesian Information Criterion (ABIC) and bootstrapped likelihood ratio test. The classes obtained were also inspected for clinical meaningfulness.

90-day mortality was compared by the log rank test.

Analyses were performed using SPSS software system version 24.0 (IBM, Armonk, NY), with the FUZZY extension except for LCA analyses, which were conducted using Mplus (version 8.14; Muthén & Muthén, Los Angeles, CA), with data preparation in R version 3.4.0 (The R Foundation for Statistical Computing).

### Ethics approval and consent to participate

The study was approved by the Lund University Ethical Review Board, decision number 2015/285. The Lund University Ethics Review Board waived the requirement for informed consent.

## Results

### Patient characteristics

**Figure 1:**
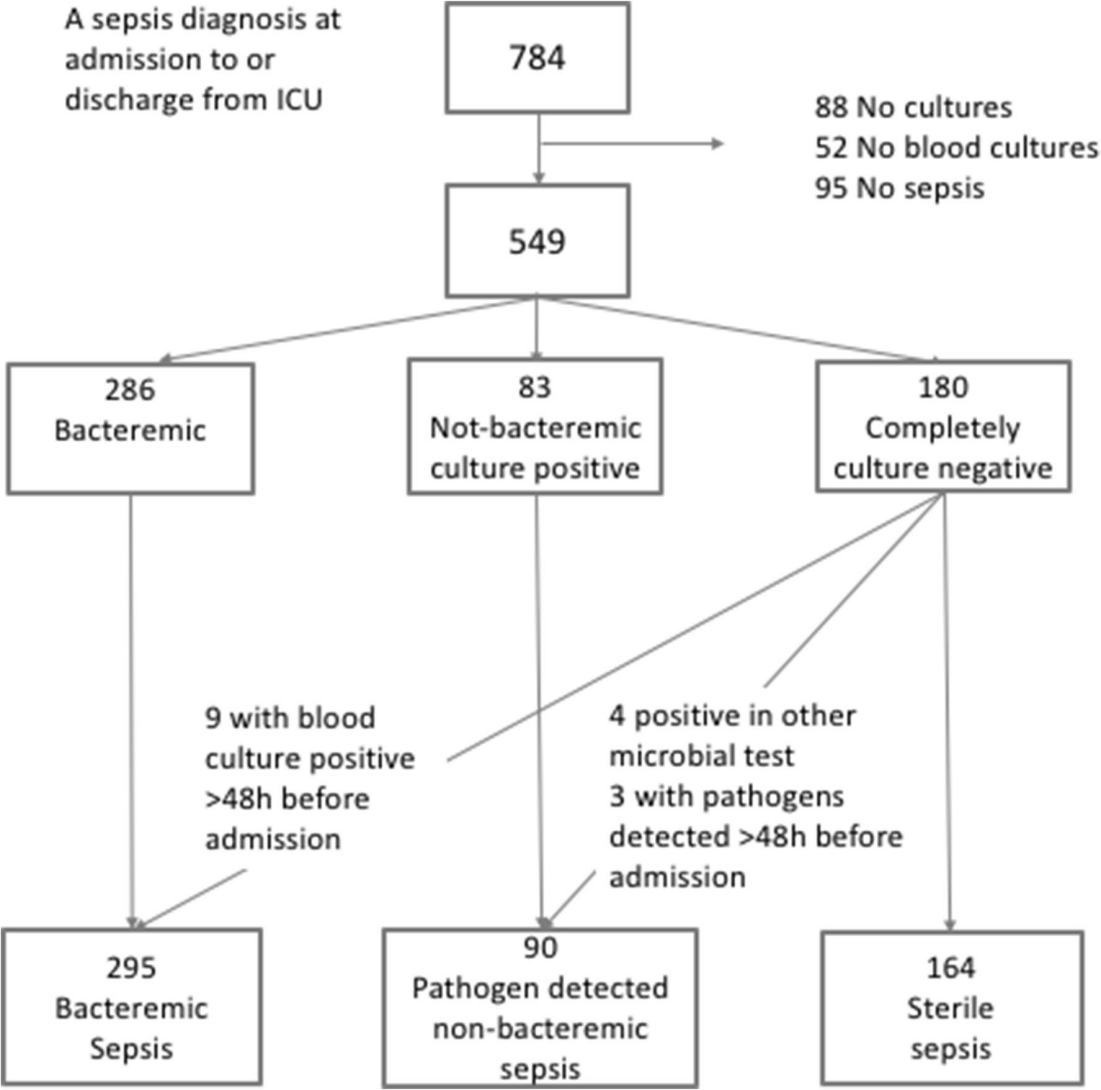
Flow chart.

A flow chart of patients is presented in Fig. 1. Over the eight study years, 784 patients were treated in the ICU and received a sepsis or septic shock diagnosis. Microbiological samples were not obtained within the predefined time interval in 88 excluded patients. Additionally 52 patients lacked blood cultures and were excluded. Ninety-five patients were excluded at the medical chart review due to absence of sepsis-3 diagnosis and 549 patients were finally included in the study. Blood cultures were positive in 286 of the patients, 83 patients had pathogen-detected but non-bacteremic sepsis. One hundred and eighty patients had sterile sepsis without positive cultures. Four sterile patients were positive in polymerase chain reaction assay (PCR); 2 were positive for legionella and 1 for influenza in respiratory tract samples and 1 for meningococci in cerebrospinal fluid and were reclassified as pathogen-detected but non-bacteremic sepsis. Additionally, nine patients with positive blood culture and 3 patients where pathogens were detected from the site of infection, outside the 96-h time frame surrounding ICU admission, were reclassified as bacteremic and pathogen-detected but non-bacteremic, respectively. This resulted in 54% (n = 295) bacteremic sepsis, 16% (n = 90) pathogen-detected but non-bacteremic sepsis and 30% (n = 164) sterile sepsis, see Fig. 1.

**Figure 2:**
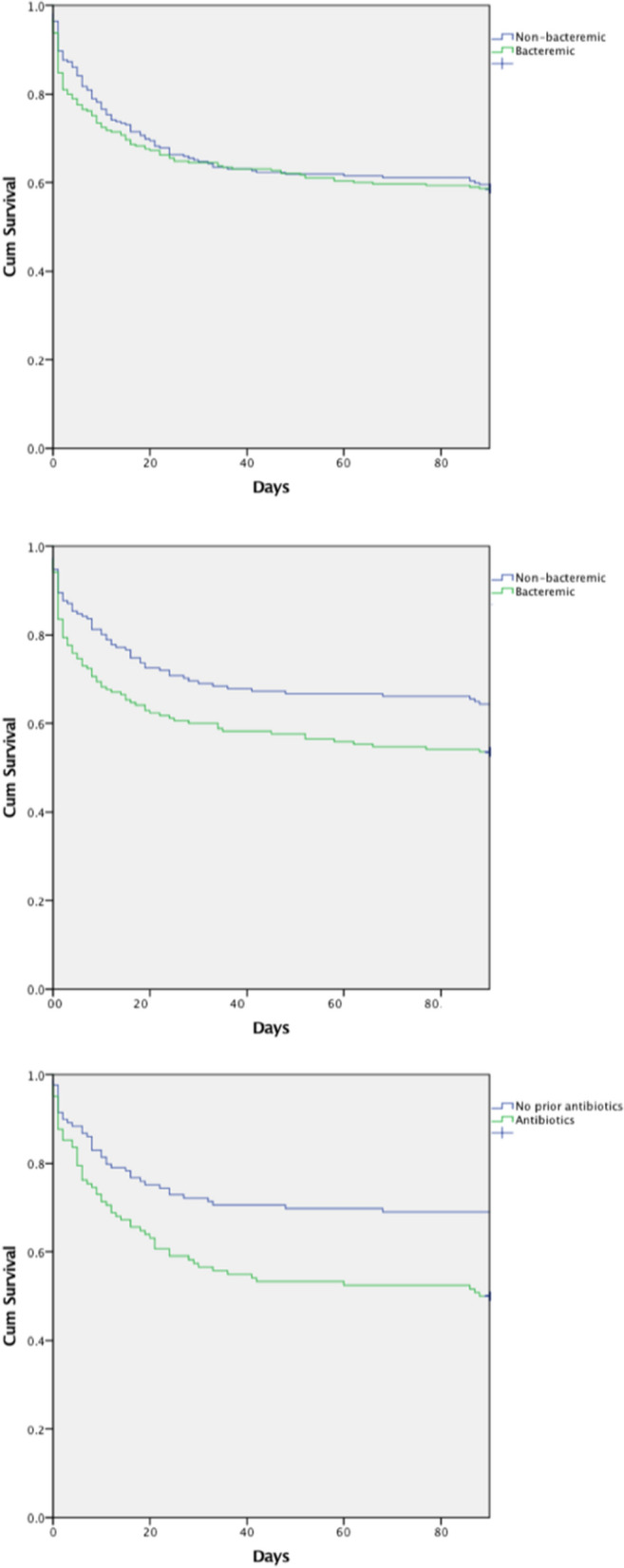
(**A**) 90-day mortality in bacteremic and non-bacteremic patients, non-adjusted cohort, showing no significant difference 42% versus 41%, *p* = 0.74. (**B**) 90-day mortality in bacteremic and non-bacteremic patients, propensity matched cohorts, showing a significant difference 47% versus 36% *p* = 0.04. (**C**) 90-day mortality in non-bacteremic patients with/without prior antibiotic therapy showing a significant difference 51% versus 31%, *p* < 0.01.

There were no missing data in the primary analyses. There were no differences in mortality between the unadjusted groups of different microbiological status, Fig. 2a. For differences in demography and clinical characteristics between bacteremic and non-bacteremic patients see Tables 1 and 2.

**Table 1:** Demography and morbidity of all included patients.

		Bacteremic n = 295	Pathogen detected, non-bacteremic n = 90	Sterile n = 164	*p*	Bacteremic vs not *p*
Age, median (IQR)		67 (59–75)	66 (55–73)	67 (58–75)	0.47	0.37
Gender, female (%)		127 (43)	42 (47)	71 (43)	0.83	0.74
**Comorbidities**						
Charlson CI, median (IQR)		2 (2–3)	2 (0–3)	2 (1–2)	0.97	0.82
Liver disease (%)		16 (5)	1 (1)	2 (1)	0.03	< 0.01
Renal disease (%)		27 (9)	4 (4)	11 (7)	0.29	0.15
Diabetes mellitus (%)		62 (21)	16 (18)	34 (21)	0.82	0.77
Acute myocardial infarction (%)		38 (13)	18 (20)	28 (17)	0.19	0.09
Congestive heart failure (%)		34 (12)	8 (9)	18 (11)	0.78	0.64
Peripheral vascular disease (%)		10 (3)	6 (7)	5 (3)	0.31	0.56
Cerebrovascular accident (%)		20 (7)	9 (10)	14 (9)	0.56	0.32
Hemiplegia(%)		9 (3)	3 (3)	6 (4)	0.94	0.74
Dementia (%)		3 (1)	1 (1)	4 (3)	0.45	0.48
COPD (%)		26 (9)	11 (12)	22 (14)	0.27	0.11
Connective tissue disease (%)		23 (8)	9 (10)	12 (7)	0.74	0.83
Peptic ulcer disease (%)		13 (4)	4 (4)	5 (3)	0.76	0.61
Malignancy(%)		88 (30)	25 (28)	48 (29)	0.93	0.78
**Medication**						
Oral steroids (%)		40 (14)	17 (19)	25 (15)	0.46	0.32
Chemotherapy (%)		48 (16)	13 (14)	19 (12)	0.41	0.23
Immunomodulatory medication (%)		13 (4)	7 (8)	14 (9)	0.16	0.06
Preceding antibiotic therapy (%)		72 (24)	40 (44)	84 (52)	< 0.01	< 0.01
**Severity of disease**						
SOFA max, median (IQR)		11 (9–14)	9 (8–12)	10 (7–13)	< 0.01	< 0.01
SOFA resp max, median (IQR)		3 (2–4)	3 (3–4)	3 (3–4)	0.32	0.14
SOFA renal max, median (IQR)		2 (1–4)	1 (0–2)	1 (0–3)	< 0.01	< 0.01
SOFA liver max, median (IQR)		0 (0–2)	0 (0–1)	0 (0–1)	0.04	0.02
SOFA coag max, median (IQR)		2 (0–3)	2 (0–3)	1 (1–2)	< 0.01	< 0.01
SOFA cardiovasc max, median (IQR)		3 (1–4)	3 (1–4)	3 (1–4)	0.66	0.38
SOFA CNS max, median (IQR)		1 (0–2)	1 (0–2)	1 (0–2)	0.88	0.85
Lactate max mmol/L, median (IQR)		4.4 (2.8–7.2)	4.0 (2.6–6.5)	3.6 (2.4–5.8)	0.01	0.01
**Outcome**						
ICU mortality (%)		71 (24)	13 (14)	46 (28)	0.05	0.82
90-day mortality (%)		125 (42)	32 (36)	72 (44)	0.41	0.74

*IQR* interquartile range, *CI* Comorbidity Index, *COPD* Chronic Obstructive Pulmonary Disease, *SOFA* Sequential Organ Failure Assessment, *CNS* Central Nervous System.

**Table 2:** Infection, microbial findings and cultures of all included patients.

	Bacteremic n = 295	Pathogen detected non-bacteremic n = 90	Sterile n = 164	*p*	Bacteremic vs not *p*
**Foci**					
Respiratory tract (%)	49 (17)	27 (30)	57 (35)	< 0.01	< 0.01
Urinary tract (%)	66 (23)	7 (8)	4 (2)	< 0.01	< 0.01
Abdominal (%)	41 (14)	21 (23)	52 (32)	< 0.01	< 0.01
Postoperative (%)	17 (6)	13 (14)	20 (12)	0.01	< 0.01
CNS (%)	11 (4)	2 (2)	1 (1)	0.12	0.06
Bone & joint (%)	7 (2)	2 (2)	2 (1)	0.69	0.51
Heart (%)	10 (3)	2 (2)	3 (2)	0.59	0.43
Skin & soft tissue (%)	30 (10)	12 (13)	5 (3)	< 0.01	0.15
Other or unknown (%)	64 (22)	2 (2)	20 (12)	< 0.01	< 0.01
Nosocomial (%)	30 (10)	18 (20)	36 (22)	< 0.01	< 0.01
**Pathogens**					
*E. coli* (%)	78 (26)	10 (11)		< 0.01	
*S. aureus* (%)	30 (10)	7 (8)		0.50	
*S. pneumoniae* (%)	34 (12)	10 (11)		0.91	
Other streptococci (%)	38 (13)	16 (18)		0.24	
Other enterobacterales	26 (9)	6 (7)		0.52	
Enterococci (%)	12 (4)	2 (2)		0.54	
Meningococci (%)	3 (1)	1(1)		1.0	
*Candida* (%)	8 (3)	1 (1)		0.69	
*P. aeruginosa* (%)	11 (4)	2 (2)		0.74	
Other (%)	55 (19)	35 (39)		< 0.01	
**No of patients with cultures obtained**					
1 blood culture pair (%)	295 (100)	90 (100)	164 (100)	1.0	1.0
2 blood culture pairs (%)	258 (87)	58 (64)	108 (66)	< 0.01	< 0.01
3 blood culture pairs (%)	82 (28)	20 (22)	36 (22)	0.30	0.12
4 blood culture pairs (%)	18 (6)	5 (6)	10 (6)	0.96	0.92
Respiratory tract (%)	82 (28)	38 (42)	68 (41)	< 0.01	< 0.01
Feces (%)	33 (11)	11 (12)	11 (7)	0.05	0.23
Urine (%)	228 (77)	64 (71)	108 (66)	< 0.01	0.03
Wound (%)	88 (30)	41 (46)	28 (17)	< 0.01	0.98
Drainage (%)	8 (3)	10 (11)	9 (5)	0.02	0.02
Synovial fluid (%)	3 (1)	3 (3)	0 (0)	< 0.04	1.0
Cerebrospinal fluid (%)	20 (7)	3 (3)	5 (3)	0.19	< 0.01
**No of patients with cultures from presumed infection foci**					
Respiratory tract (%)	36 (73)	25 (93)	41 (72)	0.09	0.50
Urine (%)	61 (92)	7 (100)	3 (75)	0.33	0.86
Postoperative (%)	11 (65)	12 (92)	11 (52)	0.08	0.94
CNS (%)	11 (100)	2 (100)	1 (100)	1.0	1.0
Bone & joint (%)	4 (57)	1 (50)	2 (100)	0.97	0.55

*CNS* Central nervous system.

### Patients without preceding antibiotic therapy

In 352 patients not treated with antibiotics prior to culture sampling, 63% (n = 223) were bacteremic, 14% (n = 50) were non-bacteremic but with positive microbial samples from foci of infection, and 22% (n = 79) had sterile sepsis, see Table 2.

These patients had similar characteristics except for renal disease being more common among bacteremic patients than non-bacteremic patients, 20 versus 4 patients, respectively, *p* = 0.04. Chronic obstructive pulmonary disease (COPD) was, however, less common among bacteremic patients, 20 patients versus 21 patients, *p* = 0.04, and likewise immunomodulatory medication in 6 bacteremic patients versus 10 non-bacteremic patients, *p* = 0.03.

### Microbial samples

*Escherichia coli* was the most common pathogen detected from blood, and non-pneumococcal streptococci were the most common pathogens from other non-blood sites.

Patients from the bacteremic sepsis, pathogen-detected but non-bacteremic sepsis or sterile sepsis groups were compared for proportion of patients with microbial samples from presumed infectious foci. Generally, more microbial samples were sampled from patients with pathogens detected. When compared for proportion of patients with microbial samples from presumed infection foci, the differences between pathogen-detected and sterile sepsis did not remain for respiratory tract, urinary tract, central nervous system, bone and joints and drainage or wound for postoperative infections. A higher number of blood culture pairs were drawn in bacteremic patients, (mean 2.2, 95% CI 2.1–2.2) compared to non-bacteremic patients (mean 1.9, 95% CI 1.8–2.0) *p* < 0.01. All patients had at least one pair of blood culture drawn. Bacteremic patients had a higher proportion of a second pair of blood cultures drawn. The additional rate of positivity in the second pair of blood culture was 7.4% for the bacteremic patients. The addition of a second pair of blood culture in the non-bacteremic patients with only one pair of blood culture drawn, with an equal rate of positivity as in the bacteremic sepsis, would be equivalent to 4 more patients becoming bacteremic (Table 2).

### Outcome

Comparisons of mortality between groups of patients with positive and negative microbiological samples, respectively, are most likely vulnerable to confounders, such as that different foci of infection have both different mortalities and different frequencies of pathogen detection^26^. We address this problem with a propensity score analysis. There were no missing data on the variables in the propensity score analysis.

In the entire cohort 172 matched pairs were retrieved, for clinical characteristics in the matched groups, see Table 3. Mortalities were significantly higher among bacteremic patients as were the total SOFA score, the SOFA scores for renal function, liver function, coagulation and lactate, Fig. 2b.

**Table 3:** Demography and morbidity of the propensity matched cohort.

		Propensity matched cohort		*p*
		Bacteremic n = 172	Non bacteremic n = 172	
Age, median (IQR)		67 (59–75)	66 (55–74)	0.42
Gender, female (%)		76 (40)	84 (44)	0.41
Charlson CI, median (IQR)		1 (0–2)	1 (0–2)	0.89
Liver disease (%)		4 (2)	3 (2)	0.74
Renal disease (%)		15 (9)	9 (5)	0.20
Diabetes mellitus (%)		34 (20)	36 (21)	0.79
Cardiovascular disease (%)		41 (24)	48 (28)	0.39
Cerebral disease (%)		17 (10)	20 (12)	0.60
COPD (%)		15 (9)	22 (13)	0.22
Connective tissue disease (%)		13 (8)	15 (9)	0.69
Peptic ulcer disease (%)		10 (6)	7 (4)	0.46
Malignancy (%)		45 (26)	46 (27)	0.90
Immunosuppressant (%)		46 (27)	40 (23)	0.46
Preceding antibiotic therapy (%)		60 (35)	59 (34)	0.91
**Foci**				
Respiratory tract (%)		39 (23)	38 (22)	0.90
Urinary tract (%)		11 (6)	11 (6)	1.0
Abdominal (%)		38 (22)	40 (23)	0.80
Bone & joint (%)		5 (3)	4 (2)	0.74
Skin & soft tissue (%)		20 (12)	16 (9)	0.48
Other (%)		59 (34)	63 (37)	0.65
**Outcome**				
ICU mortality (%)		49 (29)	37 (22)	0.14
90-day mortality (%)		81 (47)	62 (36)	0.04
**Severity of disease**				
SOFA max, median (IQR)		12 (9–16)	10 (7–13)	< 0.01
SOFA resp max, median (IQR)		3 (2–4)	3 (3–4)	0.33
SOFA renal max, median (IQR)		2 (0–4)	1 (0–3)	0.02
SOFA liver max, median (IQR)		0 (0–2)	0 (0–1)	0.05
SOFA coag max, median (IQR)		2 (0–3)	1 (0–2)	< 0.01
SOFA cardiovasc max, median (IQR)		3 (1–4)	3 (1–4)	0.58
SOFA CNS max, median (IQR)		1 (0–3)	1 (0–2)	0.28
Lactate max, mmol/L, median (IQR)		4.4 (3.0–8.0)	3.8 (2.4–6.5)	< 0.01

*IQR* interquartile range, *CI* Comorbidity Index, *COPD* Chronic Obstructive Pulmonary Disease, *SOFA* Sequential Organ Failure Assessment, *CNS* Central Nervous System.

Since the matching affected the mortality, the mortality was also compared for patients who had received antibiotic therapy prior to blood cultures. Non-bacteremic patients had significantly higher mortality if they received antibiotic therapy prior to blood cultures than if they had not received antibiotic therapy, Fig. 2c.

### Latent class analysis

The following baseline variables were categorized and entered into a latent class analysis (LCA) model: gender, bacteremia, pathogen-detected but non-bacteremic or sterile sepsis, sepsis-causing pathogen, foci of infection, prior antibiotic treatment, immunomodulatory medications, nosocomial infection, fever (> 38 °C), hypothermia (< 36 °C), acidosis (pH < 7.35), p-lactate (divided in quartiles; 0–1.8, > 1.8–3, > 3–5.1, > 5.1), and CNS-, respiratory-, cardiovascular-, renal-, liver- or coagulatory organ dysfunction. 469 patients without any missing data on the baseline variables were entered into the LCA. An eight-class model or a nine-class model were the best fit for the cohort, ABIC continued to decrease until 9-classes model, Table 4. We inspected the 8- and 9-class models and the 8-class model did yield more clinically meaningful classes and hence was chosen. The representation of class membership for baseline variables are presented in Table 5. The eight classes are characterized by:

1. Nosocomial, sterile sepsis, in this group all had nosocomial infection and none had a pathogen detected.
2. Sepsis with lactic acidosis, this group had the causative pathogen detected in all patients, of those the vast majority (92%) was bacteremic. In addition, this group had the highest frequency of organ dysfunction at admission to the ICU.
3. Community-acquired, sterile sepsis, these patients often suffered from a respiratory tract infection or an abdominal infection.
4. Abdominal sepsis with pathogen detected, the infection was community-acquired.
5. Nosocomial, pathogen-detected sepsis.
6. Sepsis with Gram-positive bacteria, the infections were community-acquired, often in the respiratory tract or skin and soft tissue infections.
7. Imunosuppressed sepsis, these patients had antibiotic therapy prior to microbial sampling and were yet bacteremic.
8. Urinary tract infection sepsis, the pathogen, *Enterobacterales*, was always detected.

**Table 4:** LCA models’ fit.

	Log likelihood HO value	AIC	BIC	ABIC	Entropy	Vouong-Lo-Mendel-Rubin *p*	Adjusted Lo-Mendel-Rubin *p*	Bootstrapped likelihood ratio test *p*
1-class	− 5861.017	11,776.033	11,888.100	11,802.407				
2-classes	− 5414.615	10,939.231	11,167.514	10,992.955	0.989	< 0.0001	< 0.0001	< 0.0001
3-classes	− 5299.530	10,765.000	11,109.560	10,846.134	0.943	< 0.0001	< 0.0001	< 0.0001
4-classes	− 5217.986	10,657.972	11,118.689	10,766.397	0.957	0.6171	0.6198	< 0.0001
5-classes	− 5161.312	10,600.623	11,177.557	10,736.399	0.948	0.77	0.77	< 0.0001
6-classes	− 5103.545	10,541.090	11,234.241	10,704.216	0.956	0.76	0.76	< 0.0001
7-classes	− 5052.411	10,494.822	11,304.190	10,685.299	0.96	0.76	0.76	< 0.0001
8-classes	− 5001.904	10,449.809	11,375.393	10,667.636	0.943	0.77	0.77	< 0.0001
9-classes	− 4958.769	10,419.538	11,461.339	10,664.716	0.923	0.86	0.86	< 0.0001
10-classes	− 4924.905	10,407.810	11,565.828	10,680.338	0.927	0.81	0.81	–

*AIC* Akaike Information Criterion, *BIC* Bayesian Information Criterion, *ABIC* adjusted Bayesian Information Criterion.

**Table 5:** Selected baseline variables in the LCA.

	Class 1	Class 2	Class 3	Class 4	Class 5	Class 6	Class 7	Class 8	*p*
	Nosocomial sterile sepsis	Sepsis with lactic acidosis	Community-acquired sterile	Abdominal pathogen detected	Nosocomial pathogen detected	Gram-positive bacteria	Immuno-suppressed sepsis	Urinary tract infection sepsis	
	n = 20	n = 37	n = 130	n = 56	n = 34	n = 112	n = 20	n = 60	
Gender, female	10 (10)	15 (41)	54 (42)	26 (46)	12 (35)	47 (42)	5 (25)	30 (50)	0.59
Bacteremia	0 (0)	34 (92)	0 (0)	28 (50)	22 (65)	86 (77)	20 (100)	58 (97)	< 0.01
Pathogen-detected	0 (0)	37 (100)	5 (4)	56 (100)	34 (100)	112 (100)	20 (100)	60 (100)	< 0.01
**Foci of infection**									
Respiratory tract	0 (0)	4 (11)	56 (43)	8 (14)	0 (0)	45 (40)	0 (0)	2 (3)	< 0.01
Urinary tract	0 (0)	4 (11)	4 (3)	3 (5)	3 (9)	0 (0)	0 (0)	50 (83)	< 0.01
Abdominal	0 (0)	14 (38)	49 (38)	34 (61)	0 (0)	0 (0)	0 (0)	4 (7)	< 0.01
Postoperative	20 (100)	0 (0)	0 (0)	0 (0)	28 (82)	0 (0)	0 (0)	0 (0)	< 0.01
Skin & soft tissue	0 (0)	0 (0)	2 (2)	8 (14)	0 (0)	27 (24)	1 (5)	0 (0)	< 0.01
Nosocomial	20 (100)	3 (8)	12 (9)	2 (4)	34 (100)	0 (0)	1 (5)	0 (0)	< 0.01
Preceding antibiotics	12 (60)	9 (24)	65 (50)	25 (10)	10 (29)	23 (21)	19 (95)	4 (7)	< 0.01
Immuno-suppression	2 (10)	11 (30)	33 (25)	18 (32)	5 (15)	13 (12)	17 (85)	4 (7)	< 0.01
**Organ dysfunction**									
Respiratory	15 (73)	37 (100)	128 (98)	51 (92)	34 (100)	101 (90)	18 (90)	54 (89)	0.01
Coagulopathy	1 (5)	26 (70)	36 (28)	3 (5)	6 (18)	43 (38)	19 (95)	31 (53)	< 0.01
Liver	2 (10)	22 (59)	16 (12)	3 (5)	3 (9)	13 (12)	5 (25)	8 (13)	< 0.01
Cardiovascular	16 (80)	35 (96)	81 (62)	31 (55)	13 (38)	78 (69)	11 (55)	40 (60)	0.02
CNS	6 (30)	22 (59)	40 (31)	13 (10)	0 (0)	44 (39)	0 (0)	15 (2)	< 0.01
Acidosis	7 (35)	35 (95)	64 (49)	21 (38)	14 (41)	66 (59)	2 (10)	12 (20)	< 0.01
Lactate < 1.7	9 (45)	0 (0)	32 (25)	11 (20)	7 (21)	39 (35)	6 (30)	8 (13)	< 0.01
Lactate 1.8–3	5 (25)	1 (3)	40 (31)	14 (25)	9 (27)	27 (24)	24 (27)	6 (30)	0.09
Lactate 3.1–5.1	3 (15)	4 (11)	35 (27)	19 (34)	7 (21)	20 (18)	5 (25)	21 (35)	0.04
Lactate > 5.1	3 (15)	32 (87)	23 (18)	12 (21)	11 (32)	26 (23)	3 (15)	15 (25)	< 0.01
Fever	8 (40)	4 (11)	36 (28)	10 (18)	10 (29)	41 (37)	7 (35)	14 (23)	0.04
Hypothermia	1 (5)	12 (32)	20 (15)	5 (9)	2 (6)	10 (9)	1 (5)	5 (8)	< 0.01
*S.aureus*	0 (0)	1 (3)	1 (1)	0 (0)	4 (12)	24 (21)	3 (15)	0 (0)	< 0.01
Enterobacterales	0 (0)	15 (41)	1 (1)	10 (18)	8 (24)	3 (3)	6 (30)	55 (92)	< 0.01
Streptococci	0 (0)	0 (0)	0 (0)	2 (4)	3 (9)	77 (69)	2 (10)	0 (0)	< 0.01
*Candida*	0 (0)	1 (3)	0 (0)	0 (0)	3 (9)	0 (0)	2 (10)	0 (0)	< 0.01
Others	0 (0)	20 (54)	2 (2)	44 (79)	16 (47)	8 (7)	7 (35)	5 n	< 0.01

Number of patients with certain demographic features, foci of infection, organ dysfunction and pathogen, percentages within parenthesis.

*CNS* Central Nervous System.

**Figure 3:**
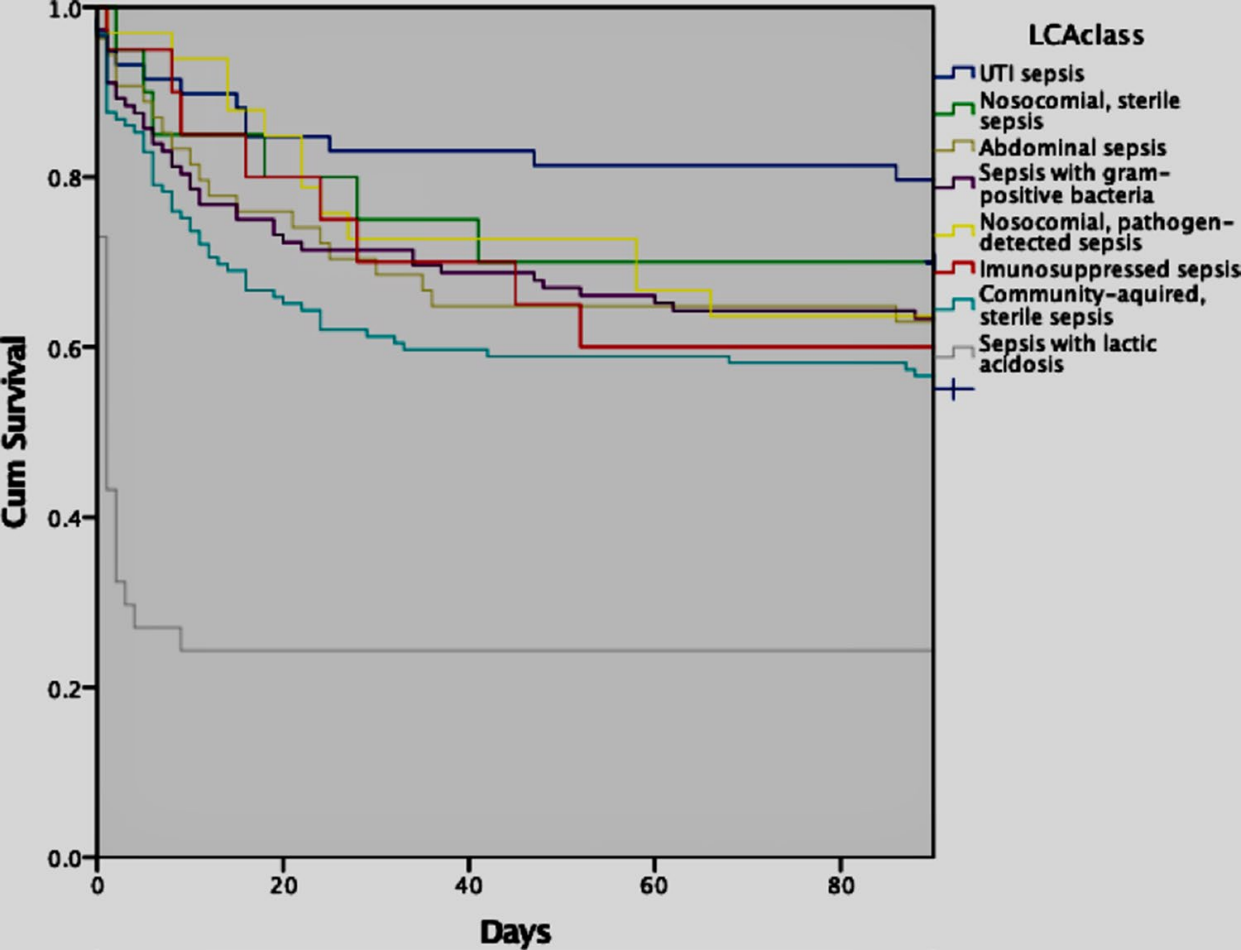
Mortality in the eight classes derived from LCA. 90-day mortality in the eight classes derived from LCA, *p* < 0.01.

90-day mortality for the different classes was compared in a Kaplan–Meier model, Fig. 3.

There was a difference in 90-days mortality between classes (*p* < 0.01) with the lowest mortality in class 8, Urinary tract infection sepsis and the highest in class 2, Sepsis with lactic acidosis.

## Discussion

The main findings in this study are that firstly, bacteremia is associated with poor outcome, and secondly that a higher percentage than previously reported of ICU patients with sepsis had positive blood cultures and other microbiological samples when analyzed with clinical chart reviewed sepsis diagnosis. Thirdly, blood culture positivity is affected by prior antibiotic treatment.

The high proportion of bacteremic patients, 54% in this study compared to 7–37% in other studies, may at least partially be explained by the manual chart review in the present study^2,3,6,8,27–29^. Two prior studies classify patients as bacteremic depending on ICD-code, which might lead to a proportion of patients being misclassified as culture-negative^9,27^. Others define sepsis as blood cultures drawn in combination with, for example two Systemic Inflammatory Response Syndrome (SIRS) criteria or ICU care, which includes other diagnoses than sepsis as well^27–29^.

We applied strict inclusion criteria of patients with at least blood cultures drawn and fulfilling an infection diagnosis and a corresponding sepsis-3 diagnosis. If we would have relied on administrative data like hospital discharge diagnosis data with *International Classification of Disease* codes (ICD) or electronic health record algorithms based on blood cultures for diagnosis, 95 (15%) patients not fulfilling infection or sepsis-3 definitions would have been included. Further, ICD-based strategies would even risk to include the 140 (18%) patients without blood cultures taken. As a poor accuracy of ICD-coding for sepsis is well documented, clinical chart reviews should be considered “gold standard” in sepsis epidemiology studies when prospective methods are not possible to use^30–33^. Results from studies based on automated electronic health record data, should be interpreted with caution even if based on large amounts of data.

Another explanation for the high proportion of bacteremic sepsis might be the high morbidity in this cohort, since bacteremic patients had even higher severity of illness and higher mortality than their non-bacteremic counterparts. The sterile sepsis proportion is similar to the numbers described in a previous prospective study on patients with septic shock where 2651 patients (31%) had sterile sepsis^10^. We also included other microbiological samples than culture, although they constituted only a small proportion of the pathogen-detected non-bacteremic sepsis group.

Sepsis is a highly heterogenous condition and different foci of infection have both different mortalities as well as different diagnostic yield of cultures and other microbiological analyses^34^. In the present study propensity score match was used to reduce baseline differences between the groups and to estimate differences in morbidity and mortality with minimal bias.

Bacteremic patients demonstrated higher mortality rate than controls in the present study.

Previous studies have demonstrated bacteremia being associated with both higher and lower mortality and to not affect mortality^6,17–22^*.* In a retrospective analysis of a prospective cohort by Komori et al., bacteremia was not associated with higher mortality when they adjusted for SOFA score^22^. We did not include scores for severity of disease in the primary analysis, as we see it as an intermediate in sepsis progression rather than a confounder.

We demonstrate that preceding antibiotic therapy is a confounder affecting mortality in non-bacteremic patients. Possibly, this could be attributed to a proportion of non-bacteremic sepsis that might in fact be bacteremic but with loss of pathogen detection in samples due to antibiotic therapy preceding culture sampling.

Higher mortality in bacteremic sepsis and in non-bacteremic sepsis with prior antibiotic treatment can also be indicative of bacterial load in blood being associated with sepsis severity, which previously has been demonstrated for isolated pathogens^35–38^.

Still, 30% of the patients had sterile sepsis, i.e. negative in all microbiology samples, with a mortality of 44%. With the high incidence of sepsis and the emerging antimicrobial resistance, sterile sepsis is a substantial cause of morbidity which needs to be examined further. Sterile sepsis has been speculated to depend on misdiagnosis of other conditions, preceding antibiotic therapy, insufficient culture sampling, handling or culture techniques^6^. The clinical chart review minimizes the misdiagnosis of other conditions and Kethireddy et al., demonstrated an increase in mortality with delayed antimicrobial therapy in both groups, indicating bacterial cause^10^.

Our results suggest sterile and non-bacteremic sepsis to partially depend on prior antibiotic therapy. The proportion of sterile sepsis patients decreased from 43% with prior antibiotic therapy to 22% sterile sepsis patients without prior antibiotic therapy, and there was a similar decrease from 63% bacteremic patients without prior antibiotic therapy to 37% bacteremic sepsis patients with prior antibiotic therapy. Thus, antibiotic therapy seems to be a predictor for culture-negative sepsis. This is in contrast to Previsdomini et al., although they also noticed this trend but was possibly limited by a smaller sample size^29^.

The LCA offers clinical subphenotypes in a classification, that might not be identified and its impact might not be tested assuming accepted standards. Neither the site nor the microbiology alone distinguished the classes, yet the combinations together with where the infection was acquired, immunosuppression and lactic acidosis were important for class distinction but also for outcome (Table 5). This finding is in accordance with the propensity score analysis (Table 3). When defining subphenotypes in sepsis, pathogen detection in microbial samples seems to have a high impact on probability of belonging to a class. This has previously been demonstrated for septic shock, while it was not a variable in a LCA used for ARDS and might have an impact in staging models like the PIRO system (predisposition, insult, response, organ dysfunction)^39–41^.

The strengths of this study are; firstly, the considering of the effect of antibiotic therapy prior to collection of microbiological samples, since it proved to be a confounder affecting both pathogen detection and mortality. Secondly, the inclusion of other microbial samples than cultures (e.g. PCR) and thirdly, the data on and the high number of microbial samples collected, elucidate a proportion of non-bacteremic sepsis and demonstrate sterile sepsis not to be due to lack of samples for pathogen detection. Further, the results do not solely rely on administrative or microbial data. All infection diagnoses and all data from microbial analysis have been reviewed by an infectious disease specialist. The proportion sterile sepsis might be underestimated since patients without an identified pathogen are less likely to obtain an infection diagnosis.

The major weakness of this study is the retrospective design. As microbiological samples were ordered as part of clinical workup, insufficient culture sampling might contribute to the microbiology negative cohorts. Fewer number of samples were drawn from blood, urine and wounds for microbiology-negative patients and fewer respiratory tract samples were withdrawn from bacteremic patients, although when cultures were compared to presumed infectious foci, there were no significant differences in ratio. Also, the handling of microbiological samples and laboratory techniques were part of clinical practice and out of study control. Other weaknesses of the study are the relatively small size and the single center conduct. We have not evaluated the effect of intra- and inter-observer variation in extracting data, to minimize these variations, clear definitions were applied^1,25^.

The classes created by the LCA were created out of, and limited to observable characteristics of the variables entered.

## Conclusions

In summary, bacteremia as well as preceding antibiotic treatment in non-bacteremic patients are related to poor outcome. Bacteremia is more common than previously described in sepsis, when a clinical chart review is used as gold-standard. A substantial portion of sepsis patients that remain microbiology-negative cannot be attributed to misdiagnosis or preceding antibiotic treatment. Distinction of subphenotypes might be useful and we demonstrated microbiological-negativity to be an important factor of a subphenotype for sepsis, with potential to reduce population and treatment heterogeneity for sepsis and improve the outcome of trials.

## Supplementary Information

Supplementary Information

## Data Availability

Anonymised data from the study is available upon reasonable request.

## Abbreviations

ABIC: Adjusted Bayesian information criterion
AIC: Akaike information criterion
BIC: Bayesian information criterion
CI: Confidence interval
COPD: Chronic obstructive pulmonary disease
ICD: International classification of disease
ICU: Intensive care unit
IQR: Interquartile range
LCA: Latent class analysis
PCR: Polymerase chain reaction
PIRO: Predisposition, insult, response, organ dysfunction
SOFA: Sequential organ failure assessment

## References

[1] Singer M, Deutschman CS, Seymour CW, Shankar-Hari M, Annane D, Bauer M (2016). The third international consensus definitions for sepsis and septic shock (Sepsis-3). JAMA.

[2] Phua J, Ngerng W, See K, Tay C, Kiong T, Lim H (2013). Characteristics and outcomes of culture-negative versus culture-positive severe sepsis. Crit. Care (London, England)..

[3] Rhee C, Dantes R, Epstein L, Murphy DJ, Seymour CW, Iwashyna TJ (2017). Incidence and trends of sepsis in US hospitals using clinical vs claims data, 2009–2014. JAMA.

[4] Mellhammar L, Wullt S, Lindberg A, Lanbeck P, Christensson B, Linder A (2016). Sepsis incidence: a population-based study. Open Forum Infect. Dis..

[5] Todorovic Markovic M, Pedersen C, Gottfredsson M, Todorovic Mitic M, Gaini S (2019). Epidemiology of community-acquired sepsis in the Faroe Islands—a prospective observational study. Infect. Dis. (London, England).

[6] Nannan Panday RS, Lammers EMJ, Alam N, Nanayakkara PWB (2019). An overview of positive cultures and clinical outcomes in septic patients: a sub-analysis of the Prehospital Antibiotics Against Sepsis (PHANTASi) trial. Crit. Care (London, England)..

[7] Ljungstrom L, Andersson R, Jacobsson G (2019). Incidences of community onset severe sepsis, Sepsis-3 sepsis, and bacteremia in Sweden—a prospective population-based study. PLoS ONE.

[8] Vincent JL, Sakr Y, Sprung CL, Ranieri VM, Reinhart K, Gerlach H (2006). Sepsis in European intensive care units: results of the SOAP study. Crit. Care Med..

[9] Gupta S, Sakhuja A, Kumar G, McGrath E, Nanchal RS, Kashani KB (2016). Culture-negative severe sepsis: nationwide trends and outcomes. Chest.

[10] Kethireddy S, Bilgili B, Sees A, Kirchner HL, Ofoma UR, Light RB (2018). Culture-negative septic shock compared with culture-positive septic shock: a retrospective cohort study. Crit. Care Med..

[11] Cheng B, Li Z, Wang J, Xie G, Liu X, Xu Z (2017). Comparison of the performance between sepsis-1 and sepsis-3 in ICUs in China: a retrospective multicenter study. Shock (Augusta, Ga).

[12] Heffner AC, Horton JM, Marchick MR, Jones AE (2010). Etiology of illness in patients with severe sepsis admitted to the hospital from the emergency department. Clin. Infect. Dis. Off. Publ. Infect. Dis. Soc. Am..

[13] Seymour CW, Kahn JM, Martin-Gill C, Callaway CW, Yealy DM, Scales D (2017). Delays from first medical contact to antibiotic administration for sepsis. Crit. Care Med..

[14] Ferrer R, Martin-Loeches I, Phillips G, Osborn TM, Townsend S, Dellinger RP (2014). Empiric antibiotic treatment reduces mortality in severe sepsis and septic shock from the first hour: results from a guideline-based performance improvement program. Crit. Care Med..

[15] Garnacho-Montero J, Gutierrez-Pizarraya A, Escoresca-Ortega A, Fernandez-Delgado E, Lopez-Sanchez JM (2015). Adequate antibiotic therapy prior to ICU admission in patients with severe sepsis and septic shock reduces hospital mortality. Crit. Care (London, England).

[16] Rhodes A, Evans LE, Alhazzani W, Levy MM, Antonelli M, Ferrer R (2017). Surviving sepsis campaign: international guidelines for management of sepsis and septic shock: 2016. Intensive Care Med..

[17] Zahar JR, Timsit JF, Garrouste-Orgeas M, Francais A, Vesin A, Descorps-Declere A (2011). Outcomes in severe sepsis and patients with septic shock: pathogen species and infection sites are not associated with mortality. Crit. Care Med..

[18] Artero A, Inglada L, Gomez-Belda A, Capdevila JA, Diez LF, Arca A (2018). The clinical impact of bacteremia on outcomes in elderly patients with pyelonephritis or urinary sepsis: a prospective multicenter study. PLoS ONE.

[19] Hsu CY, Fang HC, Chou KJ, Chen CL, Lee PT, Chung HM (2006). The clinical impact of bacteremia in complicated acute pyelonephritis. Am. J. Med. Sci..

[20] Brooks D, Smith A, Young D, Fulton R, Booth MG (2016). Mortality in intensive care: the impact of bacteremia and the utility of systemic inflammatory response syndrome. Am. J. Infect. Control.

[21] Laupland KB, Davies HD, Church DL, Louie TJ, Dool JS, Zygun DA (2004). Bloodstream infection-associated sepsis and septic shock in critically ill adults: a population-based study. Infection.

[22] Komori A, Abe T, Kushimoto S, Ogura H, Shiraishi A, Saitoh D (2020). Characteristics and outcomes of bacteremia among ICU-admitted patients with severe sepsis. Sci. Rep..

[23] Gotts JE, Matthay MA (2016). Sepsis: pathophysiology and clinical management. BMJ (Clinical research ed).

[24] Prescott HC, Calfee CS, Thompson BT, Angus DC, Liu VX (2016). Toward smarter lumping and smarter splitting: rethinking strategies for sepsis and acute respiratory distress syndrome clinical trial design. Am. J. Respir. Crit. Care Med..

[25] Calandra T, Cohen J (2005). The international sepsis forum consensus conference on definitions of infection in the intensive care unit. Crit. Care Med..

[26] Weinstein MP, Towns ML, Quartey SM, Mirrett S, Reimer LG, Parmigiani G (1997). The clinical significance of positive blood cultures in the 1990s: a prospective comprehensive evaluation of the microbiology, epidemiology, and outcome of bacteremia and fungemia in adults. Clin. Infect. Dis. Off. Publ. Infect. Dis. Soc. Am..

[27] Armstrong-Briley D, Hozhabri NS, Armstrong K, Puthottile J, Benavides R, Beal S (2015). Comparison of length of stay and outcomes of patients with positive versus negative blood culture results. Proceedings (Baylor University Medical Center).

[28] Sigakis MJG, Jewell E, Maile MD, Cinti SK, Bateman BT, Engoren M (2019). Culture-negative and culture-positive sepsis: a comparison of characteristics and outcomes. Anesth. Analg..

[29] Previsdomini M, Gini M, Cerutti B, Dolina M, Perren A (2012). Predictors of positive blood cultures in critically ill patients: a retrospective evaluation. Croat. Med. J..

[30] Gaieski DF, Edwards JM, Kallan MJ, Carr BG (2013). Benchmarking the incidence and mortality of severe sepsis in the United States. Crit. Care Med..

[31] Wilhelms SB, Walther SM, Huss F, Sjoberg F (2017). Severe sepsis in the ICU is often missing in hospital discharge codes. Acta Anaesthesiol. Scand..

[32] Wilhelms SB, Huss FR, Granath G, Sjoberg F (2010). Assessment of incidence of severe sepsis in Sweden using different ways of abstracting International Classification of Diseases codes: difficulties with methods and interpretation of results. Crit. Care Med..

[33] Johansson, D., Ekstrom, H., Beronius, E. & Rasmussen, M. [Systematic medical record review in Skane. Diagnostic codes were often wrong in severe sepsis and septic shock]. Lakartidningen. 2015;112. 26371482

[34] Laupland KB, Zygun DA, Davies HD, Church DL, Louie TJ, Doig CJ (2002). Population-based assessment of intensive care unit-acquired bloodstream infections in adults: Incidence, risk factors, and associated mortality rate. Crit. Care Med..

[35] Ziegler I, Cajander S, Rasmussen G, Ennefors T, Molling P, Stralin K (2019). High nuc DNA load in whole blood is associated with sepsis, mortality and immune dysregulation in Staphylococcus aureus bacteraemia. Infect. Dis. (London, England).

[36] Rello J, Lisboa T, Lujan M, Gallego M, Kee C, Kay I (2009). Severity of pneumococcal pneumonia associated with genomic bacterial load. Chest.

[37] Hackett SJ, Guiver M, Marsh J, Sills JA, Thomson AP, Kaczmarski EB (2002). Meningococcal bacterial DNA load at presentation correlates with disease severity. Arch. Dis. Child..

[38] Marra AR, Edmond MB, Forbes BA, Wenzel RP, Bearman GM (2006). Time to blood culture positivity as a predictor of clinical outcome of Staphylococcus aureus bloodstream infection. J. Clin. Microbiol..

[39] Calfee CS, Delucchi K, Parsons PE, Thompson BT, Ware LB, Matthay MA (2014). Subphenotypes in acute respiratory distress syndrome: latent class analysis of data from two randomised controlled trials. Lancet Respir. Med..

[40] Gardlund B, Dmitrieva NO, Pieper CF, Finfer S, Marshall JC, Taylor TB (2018). Six subphenotypes in septic shock: latent class analysis of the PROWESS Shock study. J. Crit. Care.

[41] Levy MM, Fink MP, Marshall JC, Abraham E, Angus D, Cook D (2003). 2001 SCCM/ESICM/ACCP/ATS/SIS international sepsis definitions conference. Intensive Care Med..

