# Supplementary material for: Bacteremic sepsis leads to higher mortality when adjusting for confounders with propensity score matching
Source: Sci Rep. 2021 Mar 26;11:6972. doi: 10.1038/s41598-021-86346-4 (PMC7998031; doi:10.1038/s41598-021-86346-4)
Supplement: Supplementary file 1 — Supplementary Information [file 41598_2021_86346_MOESM1_ESM.docx]

Supplementary information.

Bacteremic sepsis leads to higher mortality when adjusting for confounders

with propensity score matching - Lisa Mellhammar, Fredrik Kahn, Caroline Whitlow,

Thomas Kander, Bertil Christensson, Adam Linder

Sepsis definition

Sepsis is a life-threatening organ dysfunction caused by a dysregulated host response to infection.

An acute increase by 2 or more Sequential Organ Failure Assessment (SOFA) points represents organ dysfunction and along with infection is defined as sepsis.

Supplementary table. The SOFA score

| **Organ System** | **Score** |  |  |  |  |
| --- | --- | --- | --- | --- | --- |
|  | **0** | **1** | **2** | **3** | **4** |
| **Respiration**  PaO_2_/FiO_2_, mmHg | ≥ 400 | < 400 | < 300 | < 200 with respiratory support | < 100 with respiratory support |
| **Coagulation**  Platelets x10^3^/µL | ≥ 150 | 150 - 101 | 100 - 51 | 50 -21 | < 20 |
| **Liver**  Bilirubin, µmol/L | < 20 | 20-32 | 22-101 | 102-204 | >204 |
| **Cardiovascular** | MAP ≥ 70 mmHg | MAP <70 mmHg | Catechol-amine^a^ | Catechol-amine^b^ | Catechol-amine^c^ |
| **Central nervous system,** GCS^d^ | 15 | 13-14 | 10-12 | 6-9 | <6 |
| **Renal**  Creatinine, µmol/L  Urine output, mL/d | <110 | 110-170 | 171-299 | 300-440  <500 | > 440  < 200 |

^a^ Dopamine <5 or dobutamine 1µg/kg/min for at least 1 hour

^b^ Dopamine 5.1-15 or epinephrine ≤ 0.1 or norepinephrine ≤ 0.1µg/kg/min for at least 1 hour

^c^ Dopamine >15 or epinephrine or > 0.1 or norepinephrine >0.1µg/kg/min for at least 1 hour

^d^ Glascow Coma Scale

MAP= Mean arterial pressure
